# Key factors in a rigorous longitudinal image-based assessment of retinopathy of prematurity

**DOI:** 10.1038/s41598-021-84723-7

**Published:** 2021-03-08

**Authors:** Tatiana R. Rosenblatt, Marco H. Ji, Daniel Vail, Cassie A. Ludwig, Ahmad Al-Moujahed, Malini Veerappan Pasricha, Natalia F. Callaway, Jochen Kumm, Darius M. Moshfeghi

**Affiliations:** 1 Department of Ophthalmology, Byers Eye Institute, Stanford School of Medicine, 2452 Watson Court, Palo Alto, CA 94303 USA; 2 Retina Service, Department of Ophthalmology, Massachusetts Eye and Ear, Harvard Medical School, Boston, MA 02114 USA

**Keywords:** Diseases, Medical research

## Abstract

To describe a database of longitudinally graded telemedicine retinal images to be used as a comparator for future studies assessing grader recall bias and ability to detect typical progression (e.g. International Classification of Retinopathy of Prematurity (ICROP) stages) as well as incremental changes in retinopathy of prematurity (ROP). Cohort comprised of retinal images from 84 eyes of 42 patients who were sequentially screened for ROP over 6 consecutive weeks in a telemedicine program and then followed to vascular maturation or treatment, and then disease stabilization. De-identified retinal images across the 6 weekly exams (2520 total images) were graded by an ROP expert based on whether ROP had improved, worsened, or stayed the same compared to the prior week’s images, corresponding to an overall clinical “gestalt” score. Subsequently, we examined which parameters might have influenced the examiner’s ability to detect longitudinal change; images were graded by the same ROP expert by image view (central, inferior, nasal, superior, temporal) and by retinal components (vascular tortuosity, vascular dilation, stage, hemorrhage, vessel growth), again determining if each particular retinal component or ROP in each image view had improved, worsened, or stayed the same compared to the prior week’s images. Agreement between gestalt scores and view, component, and component by view scores was assessed using percent agreement, absolute agreement, and Cohen’s weighted kappa statistic to determine if any of the hypothesized image features correlated with the ability to predict ROP disease trajectory in patients. The central view showed substantial agreement with gestalt scores (κ = 0.63), with moderate agreement in the remaining views. Of retinal components, vascular tortuosity showed the most overall agreement with gestalt (κ = 0.42–0.61), with only slight to fair agreement for all other components. This is a well-defined ROP database graded by one expert in a real-world setting in a masked fashion that correlated with the actual (remote in time) exams and known outcomes. This provides a foundation for subsequent study of telemedicine’s ability to longitudinally assess ROP disease trajectory, as well as for potential artificial intelligence approaches to retinal image grading, in order to expand patient access to timely, accurate ROP screening.

## Introduction

Retinopathy of prematurity (ROP) is a retinal vascular disease that affects premature and low-birth-weight infants. The underlying pathophysiology of the disease involves abnormal angiogenesis of the retina that can lead to irreversible vision loss if not detected and properly treated in time^1–4^. In the United States, the incidence of ROP from 1997 to 2005 was 0.17%, with an incidence of 15.58% for premature infants with an initial hospital stay greater than 28 days^5^. Additionally, it is estimated that ROP occurs in 68% of infants with a birth weight of less than 1251 g^6^. If accurate and timely screening for ROP followed by appropriate management does not occur, permanent visual impairment can ensue. Despite the existence of effective treatment methods, ROP remains a leading cause of childhood blindness worldwide^2,4,7,8^. As advances in medicine lead to continued improvement in neonatal survival, the population at risk for ROP increases, raising the need for effective screening methods capable of meeting expanding screening demands^8,9^.

Although the current gold standard for diagnosis of ROP is bedside binocular indirect ophthalmoscopy, the increasing number of at-risk infants requiring screening and a lack of sufficient ROP experts, both nationally and around the globe, has created a significant barrier to timely screening and treatment of ROP^10–12^. Telemedicine assessment of retinal photographs is an emerging method for ROP screening that has been shown to be diagnostically accurate, safe, and reliable, and has the potential to expand access to expert ROP screening^9,11,13–19^. Telemedicine assessment of retinal images removes some of the challenges of bedside exam, such as time constraints and specialist availability, allowing for a more detailed and extended review of retinal features. Furthermore, telemedicine assessment of ROP has the key benefit of allowing for longitudinal comparison of retinal images from serial exams to provide a more accurate assessment of ROP disease trajectory and treatment needs.

Prior studies of image-based ROP analysis have used traditional methods of ROP assessment based on the International Classification of ROP (zone, stage, plus, and extent)^11,19–26^, using static measurements without direct assessment of disease progression over time, which is necessary given the dynamic nature of ROP and a key factor in the determination of the timing of screening and treatment intervention^2,27,28^. The human brain is well-trained at pattern recognition, which plays a key role in telemedicine assessment since evaluators have the ability to compare multiple images side-by-side to detect changes in disease trajectory, as opposed to relying upon memory of a single image or findings of prior exams^29,30^. We aim to evaluate this concept in the context of telemedicine and the longitudinal clinical assessment of ROP disease progression.

In this study, an ROP expert graded retinal images from serial weekly patient exams, assessing ROP trajectory compared to progression from the prior week’s exam in order to longitudinally evaluate ROP progression through time. We then hypothesized which image features and retinal components may have influenced the examiner’s ability to detect longitudinal change. Using a unique, rigorous image grading system, we compared individual image views and retinal components to overall clinical assessment in order to better understand how ROP is assessed and how specific image or retinal factors correlate with overall longitudinal ROP assessment. This methods study describes a grading system that allows for assessment of ROP disease changes on a granular level, which will serve as a baseline for a subsequent prospective study to evaluate telemedicine as compared to bedside indirect ophthalmoscopy with respect to pattern recognition and the ability to longitudinally assess ROP disease trajectory. This study provides an important foundation for an enhanced understanding of telemedicine capabilities and clinical applications for ROP screening and a basis for artificial intelligence.

## Methods

Institutional Review Board (IRB 8752) approval was obtained from the Stanford University School of Medicine and the described research adhered to the tenets of the Declaration of Helsinki. Informed consent was obtained from the legal guardians of all participants for the capture, use, and publication of their retinal images.

**Figure 1:**
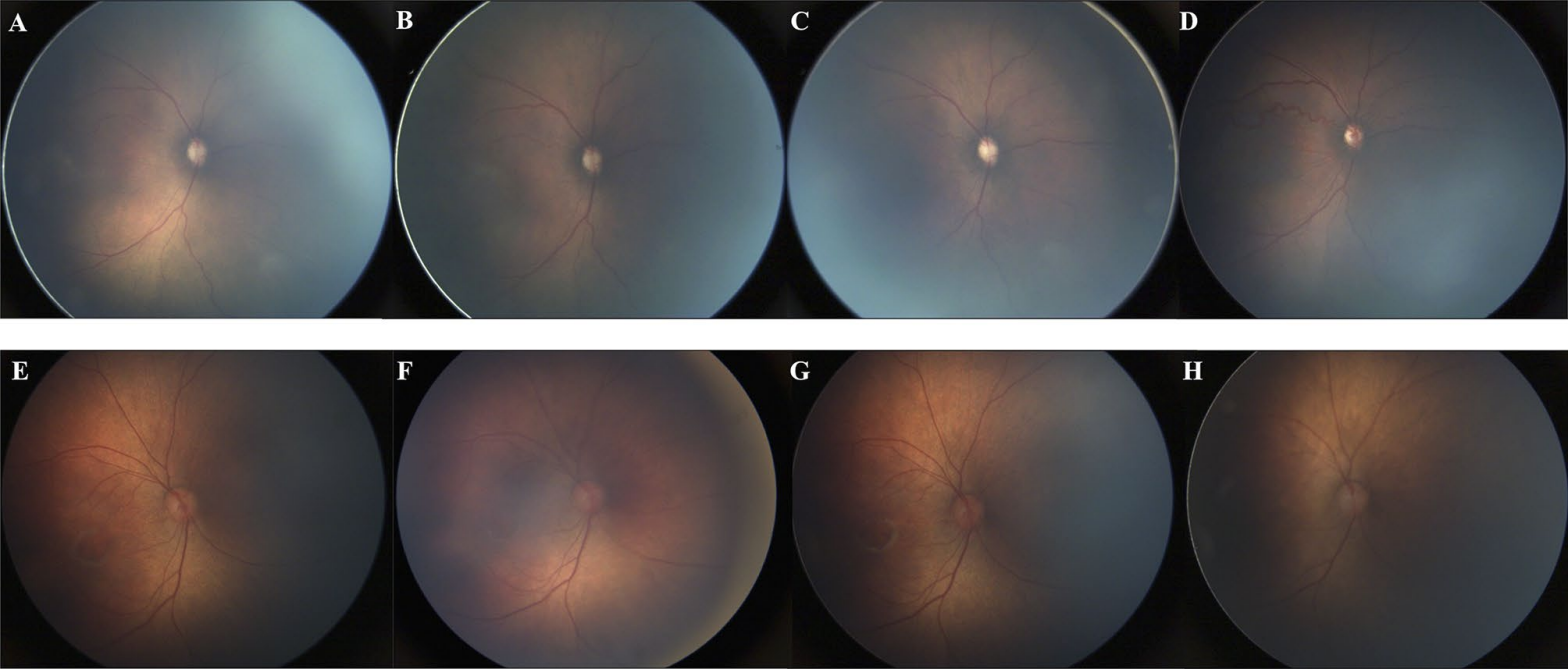
Right eye central view images from four consecutive weeks in two different patients (first patient: (**A**–**D**), second patient: (**E**–**H**)). (**A**–**D**) Continually worsening vascular tortuosity and overall worsening ROP by gestalt scores. (**E**–**H**) Stable vascular tortuosity and no overall progression of ROP by gestalt scores.

Retinal photos were obtained through the Stanford University Network for Diagnosis of Retinopathy of Prematurity (SUNDROP) database, a database of retinal images from infants at eleven participating sites who were enrolled in the SUNDROP initiative from December 1st, 2005 to November 30th, 2019. Color fundus photographs were taken by trained nursing staff using RetCam I/II/III (Natus Medical Incorporated, Pleasanton, CA) with a 130° lens. All patients’ eyes were dilated using a combination of 0.2% cyclopentolate/1% phenylephrine or 2.5% phenylephrine/1% tropicamide, administered twice 5 min apart, with patients dilated 30–60 min prior to the imaging session. Per imaging protocol, five images were taken with different orientations respective to the optic disc: central, inferior, nasal, superior, and temporal (Fig. 1).

Retinal images from 84 eyes of 42 patients were selected from the SUNDROP database for analysis in this study. Sample size was determined by calculations to assure adequate power for a subsequent prospective analysis of bedside binocular ophthalmoscopy versus telemedicine for longitudinal ROP assessment using these same images and grading system. All patients were sequentially screened for six consecutive weeks in the SUNDROP telemedicine program and then followed all the way to vascular maturation or treatment and then disease stabilization. Our cohort included a heterogeneous mix of patients who required treatment, patients  who developed disease that then spontaneously regressed, and patients  who never developed disease. Patients who received treatment for ROP and had at least 6 weeks of ROP screening images prior to their retinopathy treatment were initially selected (n = 13 patients), with the remaining 29 patients randomly selected. All patients selected had to have retinal images of each eye from at least six weekly ROP examinations.

### Image grading

A Stanford University ROP expert (D.M.M.) evaluated all images in this study. All images were de-identified with the expert evaluator blinded to infant demographic information, such as gestational age and birth weight, and treatment information. Although all patients had known anatomic outcomes in this retrospective study, all anatomic and clinical outcomes were masked to the examiner. Images were evaluated for ROP disease progression in a given week compared to the immediate prior week’s images, to assess whether ROP was getting better, worse, or staying the same. Since each patient had six weeks of exam images, this corresponded to five sets of evaluations of ROP progression (Week 1 to Week 2, Week 2 to Week 3, Week 3 to Week 4, Week 4 to Week 5, Week 5 to Week 6). In total, 2100 images were graded, plus an additional 420 images serving as the baseline Week 1 images for each eye of each patient.

#### Gestalt score

For a given week, each set of retinal images was graded based on ROP progression compared to the prior week. An overall “gestalt” score was given each week, per eye, based on overall assessment of ROP disease trajectory, with a score of + 1 given if ROP appeared to be worsening, − 1 if ROP had improved, or 0 if ROP stayed the same compared to the immediate prior week. The gestalt score was repeated four times by the same grader for each eye of each patient at the six weekly visits, with identical results with all four gradings.

#### View score

After gestalt scoring was complete for all eyes across all weeks, the examiner assessed the patient cohort again, scoring each eye with respect to a set of image and retinal features hypothesized to have potentially impacted the examiner’s initial gestalt assessment of ROP disease trajectory. With respect to image-based features, a “view score” was used wherein each eye was graded for ROP progression in each of the 5 image views relative to the optic nerve (central, inferior, nasal, superior, temporal) using the same grading scale of + 1 if ROP was worse, − 1 if ROP was better, and 0 if ROP stayed the same compared to the prior week.

#### Component score and component-by-view score

Images were also graded based on the week-to-week progression of individual retinal components. The components evaluated were the following: vascular tortuosity (VT), vascular dilation (VD), stage (ST), hemorrhage/other abnormality (H), and growth of vessels (G), the latter defined as immature retinal vascularization growing in a centrifugal pattern emanating from the optic nerve.

These five retinal components were graded using the same − 1 to + 1 scale, where − 1 meant that the specific component had improved, 0 meant that the component had stayed the same, and + 1 meant that the component had worsened compared to the prior week. These five retinal components were graded as an overall "component score" (overall VT, VD, ST, H, and G), as well as within each of the five individual retinal views ("component-by-view score"): central, inferior, nasal, superior, and temporal vascular tortuosity (CVT, IVT, NVT, SVT, TVT); central, inferior, nasal, superior, and temporal vascular dilation (CVD, IVD, NVD, SVD, TVD); central, inferior, nasal, superior, and temporal stage (CST, IST, NST, SST, TST); central, inferior, nasal, superior, and temporal hemorrhage (CH, IH, NH, SH, TH); and central, inferior, nasal, superior, and temporal growth of vessels (CG, IG, NG, SG, TG).

### Statistical analysis

All statistical analysis was conducted using R version 3.6.3 (R Foundation for Statistical Computing, Vienna, Austria). Analysis of grading variable agreement was modeled after prior studies of image-based ROP grading^19,21,27,31,32^. Image scores were analyzed by “individual” week-to-week assessments of ROP progression (scale of − 1 to + 1) and as “longitudinal” total scores across all 5 grading sessions, using the sum of each week-to-week assessment (scale − 5 to + 5) to assess overall disease trajectory. The primary outcome was agreement between gestalt scores and view scores, gestalt scores and overall component scores, and gestalt scores and component by view scores. Agreement was assessed by percent agreement (calculated as the number of times in which the two variable scores being compared were equal divided by the total number of image scores), absolute agreement, and Cohen’s weighted kappa statistic (used as a conservative estimate since it factors in the agreement that occurred by chance). As a secondary outcome measure, the Mann Whitney U test (Wilcoxon rank sum test) was used to compare average longitudinal gestalt scores in patients who eventually received treatment (treatment-warranted-ROP) and those who did not receive treatment^33–35^. Cohen’s weighted kappa statistic was interpreted using a commonly accepted scale, where 0.00–0.20 = slight, 0.21–0.40 = fair, 0.41–0.60 = moderate, 0.61–0.80 = substantial, and 0.81–1.00 = almost perfect agreement^36,37^. P values less than 0.05 were considered statistically significant.

## Results

The median weekly gestalt score for all images was + 1 (range − 1 to + 1), with a median longitudinal gestalt score, corresponding to the sum across all weeks for each eye, of + 4 (range − 1 to + 5).

### Agreement of gestalt and view scores

When assessed as individual week-to-week comparison scores, the percent agreement between scores of each view and gestalt scores ranged from 73.2 to 83.2%, absolute agreement ranged from 0.40 to 0.60, and Cohen’s weighted kappa ranged from 0.45 to 0.63, corresponding to moderate to substantial agreement (Table 1A). For all three measures of agreement, the order of views remained unchanged, with the central view showing the most agreement with gestalt scores (κ = 0.63), followed by the nasal view (κ = 0.55), temporal view (κ = 0.50), inferior view (κ = 0.46), and lastly the superior view (κ = 0.45).

**Table 1:** Agreement between gestalt and view scores.

View	% Agreement	Absolute agreement (95% CI)	Cohen’s weighted kappa (95% CI)
**A. Individual scores**^**a**^			
Central	83.2	0.60 (0.53–0.66)	0.63 (0.55–0.70)
Inferior	73.2	0.43 (0.33–0.51)	0.46 (0.38–0.53)
Nasal	79.0	0.51 (0.43–0.58)	0.55 (0.47–0.63)
Superior	75.1	0.40 (0.32–0.48)	0.45 (0.37–0.53)
Temporal	76.2	0.50 (0.42–0.57)	0.50 (0.42–0.58)
**B. Longitudinal scores**^**b**^			
Central	83.2	0.78 (0.66–0.86)	0.63 (0.52–0.74)
Inferior	44.0	0.63 (0.38–0.77)	0.49 (0.37–0.62)
Nasal	42.9	0.73 (0.54–0.83)	0.54 (0.43–0.65)
Superior	39.3	0.69 (0.53–0.80)	0.51 (0.39–0.63)
Temporal	45.2	0.73 (0.58–0.83)	0.56 (0.45–0.67)

^a^Individual scores consist of evaluation of a single week’s as compared to the prior week.

^b^Longitudinal scores consist of the sum of gestalt or view scores for each eye across all weeks.

When assessed longitudinally as the sum of weekly scores for each eye, the percent agreement ranged from 39.3 to 83.2%, absolute agreement ranged from 0.63 to 0.78, and Cohen’s weighted kappa ranged from 0.49 to 0.63 (Table 1B). The order of views in terms of most agreement to least agreement remained relatively unchanged, with the central view showing substantial agreement and the remaining four views showing moderate agreement. Furthermore, the central view had a much higher percent agreement (83.2%) with the longitudinal gestalt score than the remaining four views (39.3–45.2%).

### Agreement of gestalt and overall component scores

The five retinal components compared to the week-to-week gestalt scores demonstrated a wide range of percent agreement (26.0–77.6%), absolute agreement (0.08–0.38), and Cohen’s weighted kappa (0.008–0.47) (Table 2A). Four of the five components (vascular dilation, stage, hemorrhage, and growth of vessels) showed only slight agreement with the gestalt score (κ 0.008–0.16); however, vascular tortuosity showed moderate agreement (κ = 0.47). Vascular tortuosity and vascular dilation had the highest percent agreement, 77.6% and 62.6% respectively.

**Table 2:** Agreement between gestalt and overall component scores.

Component	% Agreement	Absolute agreement (95% CI)	Cohen’s weighted kappa (95% CI)
**A. Individual scores**^**a**^			
Vascular tortuosity	77.6	0.38 (0.34–0.43)	0.47 (0.43–0.50)
Vascular dilation	62.6	0.24 (0.20–0.28)	0.16 (0.12–0.19)
Stage	42.1	0.17 (0.12–0.23)	0.15 (0.13–0.16)
Heme	26.1	0.02 (0.001–0.04)	0.008 (0.002–0.01)
Growth of vessels	26.0	0.08 (0.03–0.13)	0.01 (0.004–0.02)
**B. Longitudinal scores**^**b**^			
Vascular tortuosity	41.0	0.67 (0.59–0.75)	0.51 (0.46–0.56)
Vascular dilation	21.7	0.29 (0.20–0.39)	0.16 (0.10–0.22)
Stage	18.3	0.25 (0.12–0.39)	0.13 (0.10–0.15)
Heme	14.5	0.02 (− 0.006 to 0.05)	0.008 (0.002–0.01)
Growth of vessels	17.4	0.02 (− 0.005 to 0.05)	0.01 (0.006–0.02)

^a^Individual scores consist of evaluation of a single week’s as compared to the prior week.

^b^Longitudinal scores consist of the sum of gestalt or view scores for each eye across all weeks.

Similar results were shown when longitudinal scores were compared, with vascular tortuosity demonstrating moderate agreement with gestalt scores (κ = 0.51) and all other components demonstrating only slight agreement (κ = 0.008–0.16) (Table 2B).

### Agreement of gestalt and component-by-view scores

When retinal components were assessed within each individual image view, Cohen’s weighted kappa showed only slight to fair agreement within all five views for vascular dilation (κ = 0.12–0.21), stage (κ = 0.003–0.32), hemorrhage (κ = − 0.004 to 0.02), and growth of vessels (κ = 0.006–0.02) (Table 3A). Vascular tortuosity demonstrated moderate agreement with gestalt scores within all five views, with the central view showing the highest agreement (κ = 0.57), followed by the temporal view (κ = 0.48), nasal view (κ = 0.44), inferior view (κ = 0.43), and lastly the superior view (κ = 0.42). Percent agreement was highest in all five views for vascular tortuosity (74.9–81.6%), followed by all five views of vascular dilation (59.7–65.1%).

**Table 3:** Agreement between gestalt and component by view scores.

Component by view	% Agreement	Absolute agreement (95% CI)	Cohen’s weighted kappa (95% CI)
**A. Individual scores**^**a**^			
Vascular tortuosity			
CVT	81.6	0.56 (0.49–0.63)	0.57 (0.50–0.65)
IVT	74.9	0.42 (0.34–0.50)	0.43 (0.35–0.51)
NVT	77.4	0.40 (0.32–0.48)	0.44 (0.35–0.53)
SVT	75.4	0.39 (0.31–0.47)	0.42 (0.33–0.51)
TVT	78.8	0.45 (0.38–0.53)	0.48 (0.39–0.56)
Vascular dilation			
CVD	65.1	0.22 (0.13–0.31)	0.21 (0.13–0.29)
IVD	62.9	0.21 (0.12–0.30)	0.19 (0.11–0.27)
NVD	62.1	0.12 (0.02–0.21)	0.13 (0.05–0.21)
SVD	59.7	0.39 (0.31–0.47)	0.12 (0.04–0.19)
TVD	63.3	0.45 (0.38–0.53)	0.15 (0.07–0.23)
Stage			
CST	25.4	0.002 (− 0.04 to 0.05)	0.003 (− 0.002 to 0.009)
IST	41.6	0.14 (− 0.012 to 0.28)	0.14 (0.10–0.18)
NST	50.2	0.31 (0.04–0.35)	0.22 (0.17–0.27)
SST	33.9	0.06 (− 0.03 to 0.16)	0.07 (0.04–0.09)
TST	59.0	0.3 (0.11–0.45)	0.32 (0.26–0.38)
Heme			
CH	25.1	0.002 (− 0.04 to 0.05)	0.002 (− 0.002 to 0.01)
IH	25.6	0.002 (− 0.04 to 0.05)	0.005 (− 0.001 to 0.01)
NH	27.9	0.02 (− 0.03 to 0.08)	0.02 (0.009–0.4)
SH	24.9	0.004 (− 0.04 to 0.05)	− 0.004 (− 0.02 to 0.01)
TH	27.1	0.02 (− 0.03 to 0.08)	0.01 (− 0.004 to 0.03)
Growth of vessels			
CG	25.4	− 0.003 (− 0.04 to 0.04)	0.01 (− 0.007 to 0.03)
IG	25.6	− 0.04 (− 0.11 to 0.04)	0.006 (− 0.01 to 0.02)
NG	26.0	− 0.02 (− 0.06 to 0.04)	0.02 (− 0.008 to 0.04)
SG	26.7	− 0.02 (− 0.06 to 0.04)	0.02 (− 0.005 to 0.04)
TH	26.2	− 0.01 (− 0.06 to 0.04)	0.01 (− 0.006 to 0.04)
**B. Longitudinal scores**^**b**^			
Vascular tortuosity			
CVT	42.9	0.82 (0.73–0.88)	0.61 (0.52–0.70)
IVT	42.9	0.68 (0.55–0.78)	0.51 (0.39–0.63)
NVT	39.3	0.68 (0.55–0.78)	0.48 (0.37–0.59)
SVT	36.9	0.66 (0.52–0.77)	0.47 (0.35–0.59)
TVT	42.9	0.66 (0.52–0.77)	0.48 (0.35–0.60)
Vascular dilation			
CVD	22.6	0.26 (0.05–0.44)	0.17 (0.04–0.30)
IVD	20.2	0.32 (0.11–0.49)	0.21 (0.08–0.34)
NVD	23.8	0.18 (− 0.034 to 0.38)	0.14 (− 0.009 to 0.28)
SVD	19.0	0.66 (0.52–0.77)	0.13 (− 0.005 to 0.26)
TVD	22.6	0.66 (0.52–0.77)	0.16 (0.02–0.30)
Stage			
CST	14.3	0.003 (− 0.05 to 0.08)	0.001 (− 0.001 to 0.004)
IST	17.9	0.2 (− 0.08 to 0.46)	0.13 (0.07–0.20)
NST	23.8	0.3 (− 0.07 to 0.57)	0.22 (0.13–0.31)
SST	14.3	0.08 (− 0.06–0.26)	0.05 (0.02–0.08)
TST	21.4	0.38 (− 0.07–0.67)	0.25 (0.17–0.34)
Heme			
CH	14.3	0.003 (− 0.05 to 0.08)	0.001 (− 0.001 to 0.004)
IH	14.3	0.003 (− 0.05 to 0.08)	0.001 (− 0.001 to 0.004)
NH	15.5	0.03 (− 0.05 to 0.12)	0.02 (− 0.006 to 0.04)
SH	14.3	0.004 (− 0.05 to 0.08)	0.003 (− 0.001 to 0.007)
TH	14.3	0.03 (− 0.05 to 0.12)	0.02 (− 0.006 to 0.03)
Growth of vessels			
CG	16.7	− 0.003 (− 0.05 to 0.07)	0.01 (− 0.003 to 0.03)
IG	17.9	− 0.04 (− 0.12 to 0.08)	0.01 (− 0.004 to 0.02)
NG	16.7	− 0.03 (− 0.09 to 0.06)	0.01 (− 0.004 to 0.03)
SG	16.7	− 0.03 (− 0.09 to 0.06)	0.01 (− 0.003 to 0.03)
TH	19.0	− 0.02 (− 0.08 to 0.06)	0.02 (0.001 to 0.03)

Central, inferior, nasal, superior, and temporal vascular tortuosity (CVT, IVT, NVT, SVT, TVT); central, inferior, nasal, superior, and temporal vascular dilation (CVD, IVD, NVD, SVD, TVD); central, inferior, nasal, superior, and temporal stage (CST, IST, NST, SST, TST); central, inferior, nasal, superior, and temporal heme (CH, IH, NH, SH, TH); and central, inferior, nasal, superior, and temporal growth of vessels (CG, IG, NG, SG, TG).

^a^Individual scores consist of evaluation of a single week’s as compared to the prior week.

^b^Longitudinal scores consist of the sum of gestalt or view scores for each eye across all weeks.

Longitudinal evaluation similarly showed the most agreement for vascular tortuosity within all five views compared to the other components, with substantial agreement demonstrated for vascular tortuosity in the central view (κ = 0.61) and moderate agreement demonstrated in the other four views (κ = 0.47–0.51) (Table 3B). All other components by view demonstrated only slight to fair agreement (κ = 0.001–0.25). All components by view showed similar absolute agreement and Cohen’s weighted kappa results, with the exception of vascular dilation in the superior and temporal views, which showed relatively low kappa statistics (SVD κ = 0.13, TVD κ = 0.16), but had relatively high absolute agreement (SVD 0.66, TVD 0.66).

### Comparison of treated and non-treated patients

Of the 42 patients included in the study, 13 patients (31.0%) eventually received treatment and 29 patients (69.0%) did not. Median longitudinal gestalt score among treated patients was + 5 (range + 4 to + 5), whereas median longitudinal gestalt score among non-treated patients was + 3 (range − 1 to + 5). When compared using the Mann Whitney U test (Wilcoxon rank sum test), treated patients had a higher average longitudinal gestalt score than non-treated patients (p < 0.0001).

## Discussion

As a whole, our cohort showed a general trend of consistent worsening of ROP from week-to-week, as evidenced by the median gestalt scores both weekly (+ 1) and longitudinal across all weeks (+ 4). Patients who ultimately received treatment had significantly higher gestalt scores, suggesting worse ROP than those who did not receive treatment and serving as a clinical validation of our gestalt score.

Although Cohen’s weighted kappa statistics were similar for individual week-to-week assessment and longitudinal assessment, absolute agreement generally increased, especially for the view scores and for vascular tortuosity, both overall and within each image view. This suggests a potential stronger correlation between individual image features and overall clinical ROP assessment when disease trajectory is assessed over a longer period of time, with general disease trends accurately captured over time despite potential mismatches in scores for an individual week.

The central image view showed the strongest agreement with the gestalt score, with substantial agreement demonstrated both for individual week-to-week scores and longitudinal assessment. After the central view, the temporal and nasal views had the next highest agreement, which is consistent with the fact that ROP tends to develop in the horizontal retinal (nasal, central, and temporal regions), often sparing the superior and inferior regions^38^. Given that no individual view showed near-perfect agreement with the gestalt score, our results suggest that analysis of all five views together, rather than analysis of only a single image, provided enhanced information that contributed to the overall clinical assessment of ROP progression by our grader. This is consistent with prior studies that have shown progressively increasing sensitivity to detect referral-warranted ROP when a single image view versus three views versus five views were assessed^22^.

Many prior studies of image-based grading of ROP have focused on diagnosis of “plus” disease, which is determined by the presence of abnormal arterial tortuosity and venous dilation to a greater degree than a standardized image, and is often the predominant factor that determines whether treatment is warranted^20,27,28,31,39,40^. However, the specific degree of vascular tortuosity and dilation indicative of a diagnosis of plus disease is difficult to quantify and varies greatly among practitioners^27^. While vascular tortuosity had by far the strongest agreement with clinical gestalt of any of the individual retinal components assessed in this study, with moderate agreement shown both overall and when analyzed within each view, vascular dilation only showed slight to fair agreement by Cohen’s weighted kappa and absolute agreement. Additionally, stage, which assesses retina and blood vessel changes at the border (“demarcation line”) of the vascularized and avascular retina, is often included as one of the key assessments of ROP^11,19–21,26^. However, our results showed only slight to fair agreement of stage with the gestalt scores, suggesting that other factors had a stronger influence on the overall clinical assessment of ROP progression by the grader in this study.

Limitations of this study include variations in image quality, typically due to varying degrees of patient cooperation as is often the case with assessment of actual patients. Poorer image quality in a specific view could be compensated for in the gestalt score by better quality images in the remaining views but could have disproportionately affected the analysis of image view agreement with gestalt scores. Additionally, by simply assessing ROP disease trajectory as better, worse, or the same compared to the prior week, we were unable to evaluate the scale of change, since a score of + 1 could mean a slight worsening from the week prior or a significant worsening. However, by keeping our grading scale more general, we were able to highlight overall trends in disease progression and reduce the variation that could stem from an attempt to quantify the amount of change observed. Lastly, our study used a single ROP expert to grade the images, and therefore the score agreement results may not be applicable to other graders, as prior studies have shown the potential for systematic biases that could lead an individual grader to over or undercall ROP disease or to place a greater respective focus on particular retinal features^27^.

In conclusion, this study describes a rigorous grading approach to image-based ROP assessment used to enhance our understanding of the individual factors that may influence overall assessment of ROP progression. We found that the central image view showed the most agreement with the gestalt score, followed by the nasal and temporal views. Vascular tortuosity showed by far the strongest agreement with gestalt, with the other components of vascular dilation, stage, hemorrhage, and vessel growth showing only slight to fair agreement. Ultimately, no single component or view had near-perfect agreement with the gestalt score, suggesting that an amalgamation of factors likely underlied the expert grader's gestalt assessment of overall ROP progression. These data provide the foundation for subsequent evaluation of telemedicine compared to bedside indirect ophthalmoscopy in the longitudinal assessment of ROP disease trajectory and can serve as a basis for an artificial intelligence approach to grading retinal images in order to expand patient access to timely and accurate ROP screening.
